# Cytokine Kinetics in the First Week of Tuberculosis Therapy as a Tool to Confirm a Clinical Diagnosis and Guide Therapy

**DOI:** 10.1371/journal.pone.0129552

**Published:** 2015-06-26

**Authors:** Alice L. den Hertog, María Montero-Martín, Rachel L. Saunders, Matthew Blakiston, Sandra Menting, Jeevan B. Sherchand, Lovett Lawson, Olanrewaju Oladimeji, Saddiq T. Abdurrahman, Luis E. Cuevas, Richard M. Anthony

**Affiliations:** 1 Royal Tropical Institute (KIT), KIT Biomedical Research, Amsterdam, The Netherlands; 2 Liverpool School of Tropical Medicine, Liverpool, United Kingdom; 3 Tribhuvan University Institute of Medicine, Kathmandu, Nepal; 4 Zankli Medical Centre, Abuja, Nigeria; 5 Abuja Federal Capital Territory Tuberculosis Control Programme, Abuja, Nigeria; University of Cape Town, SOUTH AFRICA

## Abstract

**Background:**

Many patients treated for tuberculosis (TB) in low and middle income countries are treated based on clinical suspicion without bacteriological confirmation. This is often due to lack of rapid simple accurate diagnostics and low healthcare provider confidence in the predictive value of current tests. We previously reported in an animal TB model that levels of host markers rapidly change in response to treatment initiation.

**Methods:**

We assessed the potential of host biomarker kinetics of TB patients during the first two weeks of therapy to identify patients responding to treatment. Adult patients clinically diagnosed with and treated for TB, 29 in Nigeria and 24 in Nepal, were analyzed.

**Results:**

Changes in concentrations of non-specific host biomarkers, particularly IP-10, in response to the first week of anti-TB therapy were strongly associated with bacteriological confirmation of TB. A decrease in IP-10 level of >300pg/ml between 0 and 7 days of treatment identified 75% of both smear-positive and smear-negative culture positive patients and correctly excluded TB in all nine culture negative patients.

**Conclusions:**

Monitoring of early IP-10 responses to treatment could form the basis of a simplified assay and could help identify patients who were erroneously clinically diagnosed with TB or those infected with drug resistant strains on inappropriate treatment. We believe this approach may be particularly appropriate for difficult to diagnose patients, e.g. smear-negative HIV-positive, or those with extra-pulmonary TB, often treated without bacterial confirmation.

## Introduction

Tuberculosis (TB) is a major public health problem worldwide, with an estimated 9.0 million new cases each year [1]. Many patients with suspected TB initiate treatment without bacteriological confirmation [2]. This is due to the lack of simple, rapid, and sensitive diagnostics combined with poor healthcare provider confidence in the predictive value of current tests [3,4]. Sputum smear microscopy remains the most widely available diagnostic method for TB worldwide despite the roll out of DNA amplification methods [1]. Smear microscopy however has low sensitivity and does not provide information on drug susceptibility. A large number of suspected TB patients are therefore treated empirically. This lack of confirmation can lead to unnecessary anti-TB drug exposure in those who in fact have other conditions. Furthermore, those infected with drug resistant strains often initially receive treatment with an ineffectual first-line drug regimen.

Treatment monitoring is routinely performed by microscopically assessing sputum conversion after at least 2 months of treatment. This has limited sensitivity and specificity [5] and is only useful for a subset of (initially smear positive) patients. Therefore there is a desperate need for new tools for determining the effectivity of treatment in all TB patients preferably in an early stage of therapy. Serum concentrations of some cytokines correlate with TB. For example increased concentrations of Interferon-gamma (IFNγ), Interferon gamma-induced protein 10 (IP-10), Interleukin-10 (IL-10), IL-6 and IL-12p40, and Tumor necrosis factor (TNFα) have been reported at the time of diagnosis in adults with TB [6–8]. These levels generally return to normal over the course of effective therapy [6,9], and patients who fail to respond maintain high cytokine concentrations [10]. Cytokine kinetic responses have potential to monitor treatment response [11].

Cytokine/chemokine concentrations change rapidly during the first week of therapy with Rifampicin (RIF) and Isoniazid (INH) in a mouse model of TB [9]. If replicated in human TB patients, these early changes could have potential as markers of treatment effectivity, and could be useful for confirming an empirical diagnosis as well as for monitoring treatment response. We therefore investigated the potential of cytokine kinetics in patients with suspected TB during the first two weeks of therapy to confirm or refute a clinical diagnosis of TB and to identify, relatively early, patients infected with drug resistant *Mycobacterium tuberculosis* [11].

We also measured selected antibody kinetics upon treatment initiation. Although antibody titers are known not to be useful for diagnosis of TB, we hypothesized that (free) serum antibody levels may increase upon bacterial killing and consequent stimulation by degradation products, or decrease upon reduction of bacterial load.

## Material and Methods

### Patient recruitment

Fifty-four adults ≥ 18 years old with a least a two weeks history of cough and a presumptive diagnosis of pulmonary TB were enrolled between May and June 2013. Of these, 16 and 9 patients respectively were enrolled at the German-Nepal Tuberculosis Project/Nepal Anti-TB Association (GENETUP/NATA) and Tribhuvan University Teaching Hospital/Institute of Medicine in Kathmandu, Nepal, and 29 patients were enrolled at the Wuse, Maitama, and Nyanya district hospitals in Abuja, Nigeria. All patients gave written informed consent to participate, and demographic and clinical data were collected using standard questionnaires. Subjects treated for TB in the previous 2 years, those unable to attend follow up visits, and contacts of known multi-drug-resistant (MDR) TB patients were excluded. One Nepalese patient withdrew during the study and was excluded from the analysis.

All smear-positive and smear-negative patients clinically diagnosed as having TB were enrolled consecutively by the hospital staff. Patients were retrospectively assigned to three categories: (a) smear positive culture positive; (b) smear negative culture positive, and (c) smear negative culture negative.

The decision to treat the patients was based on clinical symptoms and smear microscopy, however, GeneXpert became available in Nigeria and the results also provided to staff for clinical management. Sputum specimens were cultured on solid media and in addition, were cultured on BACTEC-MGIT 960 in Nigeria. Treatment outcomes at the end of therapy were retrieved from the TB programme treatment cards 6 and 8 months after initiation of treatment. Outcomes were reported following the World Health Organization (WHO) guidelines.

Both in Nepal and Nigeria, first line therapy consisted of 2 months of Rifampicin, Isoniazid, Pyrazinamide and Ethambutol followed by 4 months rifampicin and isoniazid.

### Ethics

Ethical approval was obtained from LSTM Research Ethics Committee (Reference number 273 [6-11-E]), the Institute of Medicine Tribhuvan University in Nepal, and the Federal Capital Territory Medical Ethics Committee in Abuja, Nigeria (Reference number FHREC/2013/01/24/05-07-13).

### Serum collection

Blood samples were collected before starting anti-TB therapy (day 0) and on days 1, 3, 5, 7, and 14 after treatment initiation. In Nigeria an additional sample was collected at day 2.

Blood samples (2–5 ml) were collected in vacutainer tubes without anti-coagulants. After 30–60 minutes at room temperature, samples were centrifuged at 1,500 rpm for 15 minutes and the resulting sera was stored at -80ºC. After transportation on dry-ice to The Netherlands, samples were stored at -80ºC until analysis. Samples were blinded for all patient information except study number and sampling day until after testing.

### Cytokine assays

Samples were tested undiluted in duplicate according to the manufacturer’s guidelines using bead-based multiplex assays HCYTOMAG-60K (Merck Millipore, Merck Chemicals B.V., Amsterdam, the Netherlands) for IP-10, IFNγ, monocyte chemotactic protein 1 (MCP-1), IL-6, and TNFα, and the HCYP3MAG-63K assay (Merck Millipore) for Monokine induced by gamma interferon (MIG). Plates were read using a Magpix machine (Luminex, Austin, Texas, USA). Data analysis was performed using Milliplex analyst v5.1 software (Vigenetech, Carlisle, Massachusetts, USA).

### Antibody ELISAs

Recombinant HSP-16 (α-crystallin, Wageningen University and Research Center, Wageningen, The Netherlands) and ESAT-6 (BEI Resources, NIAID, NIH, USA) in PBS were coated at 4 μg/ml and 2 μg/ml respectively and incubated overnight at 4^°^C. Uncoated wells were included as coating control. Plates were blocked with 10% newborn goat serum plus 0.1% Tween-20 in PBS (PBSNT) for 1h at 37^°^C. Samples were incubated in duplicate in PBSNT for 30 min at 37 ^o^C at a 1/25 dilution. Samples with high titers were retested diluted up to 1/1000. A control sample was included diluted 1/25, 1/50 and 1/100 in each plate. HRPO conjugate goat α human IgG (H+L, Jackson ImmunoResearch Laboratories Inc., West Grove, PA, USA) 1/20000 in PBSNT was incubated for 1 h at 37^°^C. Detection was performed with TMB and the reaction stopped after 20 min. Plates were read at 450 nm. Washing between all steps was performed with PBS/0.05%Tween-20 (PBST).

Arbitrary units (AU) of antibodies against HSP-16 and ESAT-6 were calculated for each sample by subtracting the Optic Density (OD) of the uncoated well from the antigen coated well. To compare levels between different plates ODs were normalized to the 1/25 (set to 100 AU), 1/50 and 1/100 fold dilutions of the control sample.

### PROTOLAM rapid tests

Semi-quantitative (negative, weakly positive, positive, or strongly positive) analysis of serum antibodies was performed on samples from a representative selection of 31 culture positive and negative patients. The lateral flow test (PROTOLAM, Lionex GmbH, Brunschweig, Germany) has two lines, one detects LAM antibodies and one unspecified TB antibodies. Tests were performed according to the manufacturer’s instructions.

### Statistical analysis

Statistical testing was done using Stata 12.1. Two-sample Wilcoxon rank-sum (Mann-Whitney) tests were used to test for differences between groups. Wilcoxon matched-pairs signed-ranks test were performed for paired testing between time points. Rapid test data showed only limited variability, the interpretation of which was quite subjective and was unsuitable for statistical testing.

## Results

### Patients

A total of 53 adult patients clinically diagnosed with and treated for TB were available for analysis (Table 1). Fifteen of 24 patients in Nepal were culture-positive (65%), eight culture-negative, and one contaminated. Eleven of the 15 culture-positive patients were smear-positive and all culture-negative patients were smear-negative (Table 1). Twenty-eight of 29 (97%) patients in Nigeria were culture and or GeneXpert positive. Of these, 20 were smear-positive and eight smear-negative (Table 1). One patient in Nigeria was smear-, culture-, and GeneXpert-negative. GeneXpert RIF resistance was detected in three Nigerian patients and their isolates underwent drug susceptibility testing (DST). DST identified one patient infected with an MDR TB strain also resistant to Ethambutol and Streptomycin, one with a strain resistant to RIF and streptomycin, and one with a fully susceptible strain. One further isolate underwent DST (mono-resistant to streptomycin) on the basis of clinical suspicion of drug resistance. HIV status was available for all patients in Nigeria and 20 of 24 in Nepal. Fifteen (52%) patients in Nigeria and one (4%) in Nepal were HIV-positive. Seven patients in Nigeria were on anti-retroviral therapy (ART) prior to TB diagnosis, three started ART at the same time as anti-TB medication, for six HIV positive patients this data was not available.

**Table 1:** Patient characteristics.

	Nepal	Nigeria
Number	24^a^	29
Median age [range]	27 [19–69]	32 [18–50]
Male sex (%)	19 (79.2)	21 (72.4)
Duration of symptoms in weeks [range]	3.5 [1–52]	8 [2–40]
Duration of cough (weeks)	2.5 [0–52]	8 [2–40]
HIV positive (%)	1 (4.2), 4 NA	15 (51.7)
Smear positive (%)	12 (50)	20 (69.0)
Positive solid culture (%)	15 (62.5), 1 NA^d^	24 (82.8), 3 NA
Positive liquid culture (%)	NA	24 (82.8), 3 NA
GeneXpert MTB (%)	NA	28 (96.6)
Combined Culture and GeneXpert result^c^	15 (62.5), 1 NA^d^	28 (96.6)
GeneXpert RIF resistance (%)	NA	3 (10.3)
DST results (of 4 patients tested)	NA	1 RIF+STR resistance
		1 MDR+STR+EMB resistance
		1 STR resistance
		1 Pansusceptible
Treatment outcomes	NA	14 cured^b^
		8 treatment completed
		5 transferred out
		2 defaulted treatment
		None died

NA: Not available (for cultures this includes contamination).

a Excludes 1 withdrawn patient see Methods.

b Reported result for both RIF resistant cases.

c Number of samples positive by either or both Culture/GeneXpert.

d The patient with NA culture was Smear positive and was included in the Culture/GeneXpert positive group in the analyses.

### Serum Cytokines levels at diagnosis and follow up

**Fig 1:**
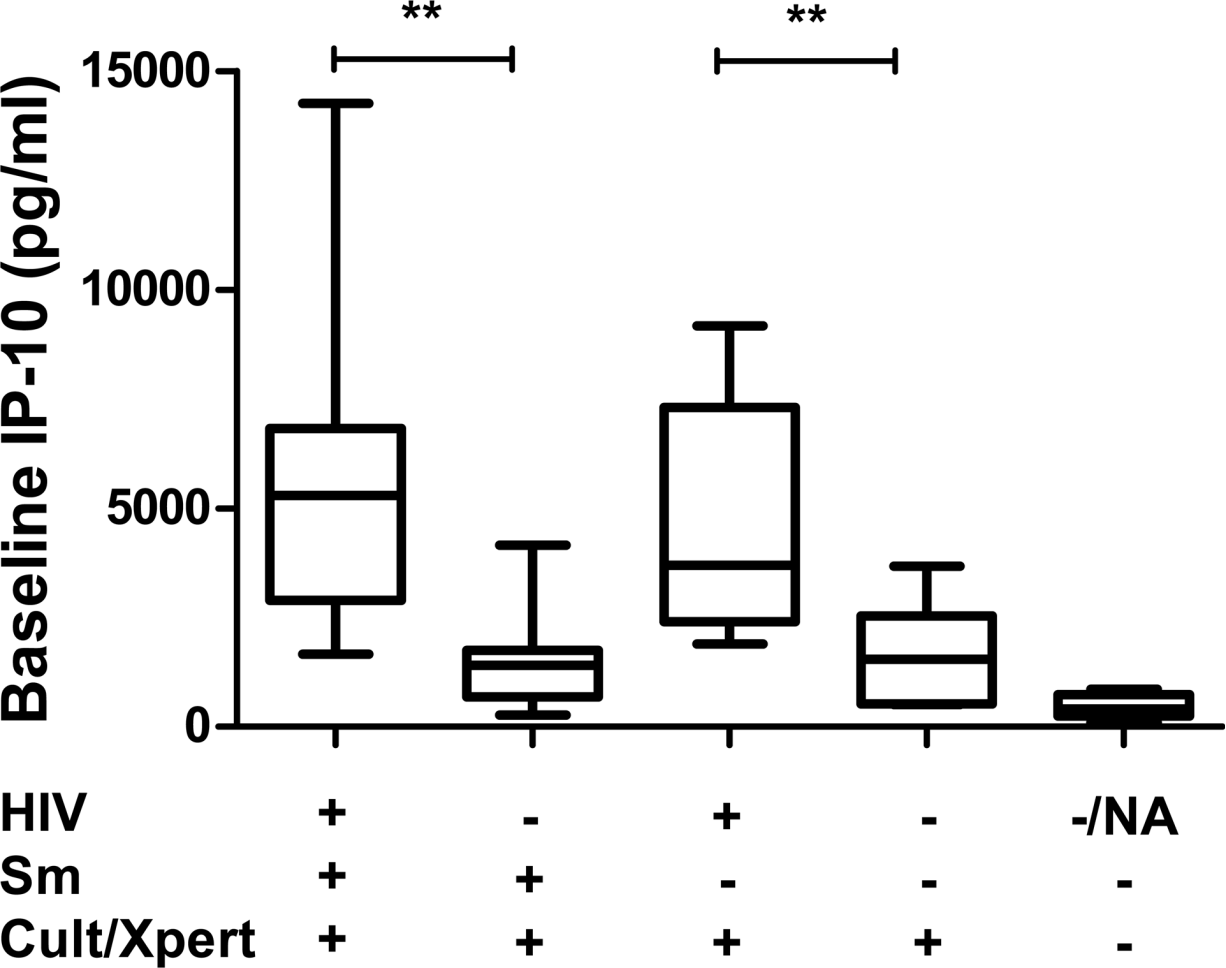
Baseline IP-10 concentrations by HIV status, Smear status, and TB culture/GeneXpert result. Box plots show medians and ranges for patients, grouped based on HIV, Smear, and culture/GeneXpert result (**p<0.01).

The median serum level of IP-10 at diagnosis (day 0) was elevated in culture/GeneXpert positive patients compared to culture/GeneXpert negative patients (p<0.001; Fig 1). Among patients with positive culture/GeneXpert results, IP-10 levels were significantly higher in HIV-positive than in HIV-negative patients (p<0.01). There was no association between smear positivity and IP-10 level on enrolment (p = 0.484; Fig 1).

**Fig 2:**
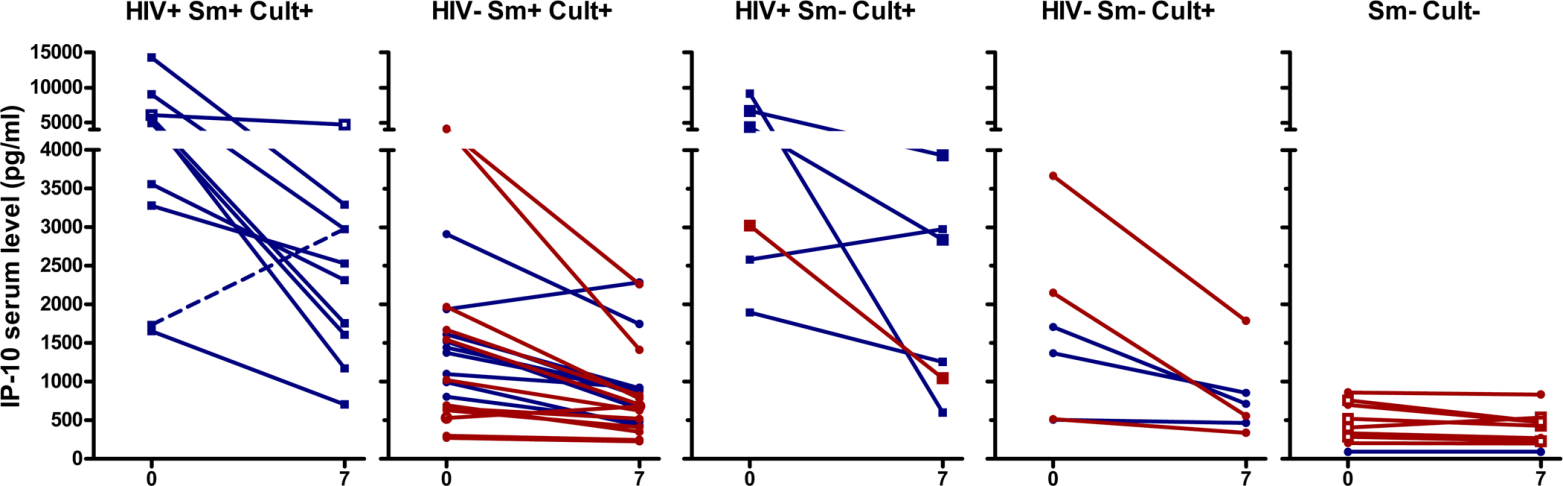
Serum IP-10 concentrations at day 0 and 7 of therapy. Each set of 2 connected points represent data from a single patient from Nigeria (blue) or Nepal (red). Panel A: IP-10 concentrations of HIV-positive, Smear (Sm) positive, and culture/Xpert (Cult) positive patients. The MDR-TB patient is shown with a dashed line. One smear-positive patient had missing culture (Open symbols). Panel B-D: IP-10 concentrations of HIV- Sm+ (B), HIV+ Sm- (C), and HIV- Sm- (D) culture/GeneXpert positive patients. E: Concentrations from Sm-Cult- patients, of which 4 had missing HIV result (open square symbols) and all others were HIV- (round symbols). IP-10 levels in culture/GeneXpert positives were significantly different between day 0 and day 7 (p<0.0001). For culture/GeneXpert negatives p>0.05 between day 0 and day 7.

The median IP-10 concentration of culture positive patients decreased during the course of therapy regardless of smear microscopy and HIV status (p<0.0001 overall, p<0.01 for each subgroup at day 7), as shown in Fig 2. For all culture positive sub-groups (HIV+/-, Smear +/-) IP-10 concentrations were significantly lower at days 5, 7, and 14 than on enrolment (Fig 2, S1 Fig). In contrast, culture-negative patients had similar IP-10 concentrations at the time of enrolment and at day 7 (p = 0.086; Fig 2). Although numbers of patients in each group are small, IP-10 responses of patients on established or simultaneously initiated ART had similar patterns and changes were likely related to anti-TB therapy not ART.

We performed ROC analyses and produced ROC curves demonstrating the ability of cytokine shifts between day 0 and day 7 of treatment to identify patients receiving appropriate treatment. For IP-10, the area under the ROC curve was 0.88 [95% CI 0.79–0.97], p<0.001(Panel A in S2 Fig). Based on the ROC curve for IP-10 response, we set a threshold of 300 pg/ml decrease between enrolment (day 0) and day 7 of treatment to differentiate between patients receiving appropriate of inappropriate treatment. This threshold of at least a decrease of 300 pg/ml at day 7confirmed appropriate treatment in 33 (75%) of 44 bacteriologically-confirmed patients and detected no response in all nine (100%) culture-negative patients (Table 2). Twenty-four (75%) of 32 smear-positive (Fig 2A and 2B) and nine (75%) of 12 smear-negative culture-positive patients (Fig 2C and 2D) had a decrease in IP-10 between day 0 and day 7 >300 pg/ml. Nine (90%) of 10 smear-positive cases co-infected with HIV had a decrease in IP-10 levels >300 pg/ml by day 7 (Fig 2A), while one patient had an increase of 1240 pg/ml. This latter patient was infected with MDR TB (Fig 2A, Dashed line) and had consistently elevated IP-10 concentrations until day 14 of the follow up (Panel A in S1 Fig).

**Table 2:** Distribution of samples with serum IP-10 decreasing >300 pg/ml between day 0 and day 7.

Culture/GeneXpert+	Smear	HIV	N	Samples (%) with IP-10 decrease of >300pg/ml
Yes	Smear+	HIV+	10	9 (90)*
		HIV-	22	15(68)
	Total smear+		32	24(75)
	Smear-	HIV+	6	5(83)
		HIV-	6	4 (67)
	Total smear-		12	9(75)
**Total**	** **	** **	**44**	**33 (75)**
No	Smear+	HIV+	0	NA
		HIV-	0	NA
	Total smear+		0	NA
	Smear-	Unknown	5	0(0)
		HIV-	4	0(0)
	Total smear-		9	0(0)
**Total**	** **	** **	**9**	**0 (0)**

* the not responding patient was confirmed MDR.

**Fig 3:**
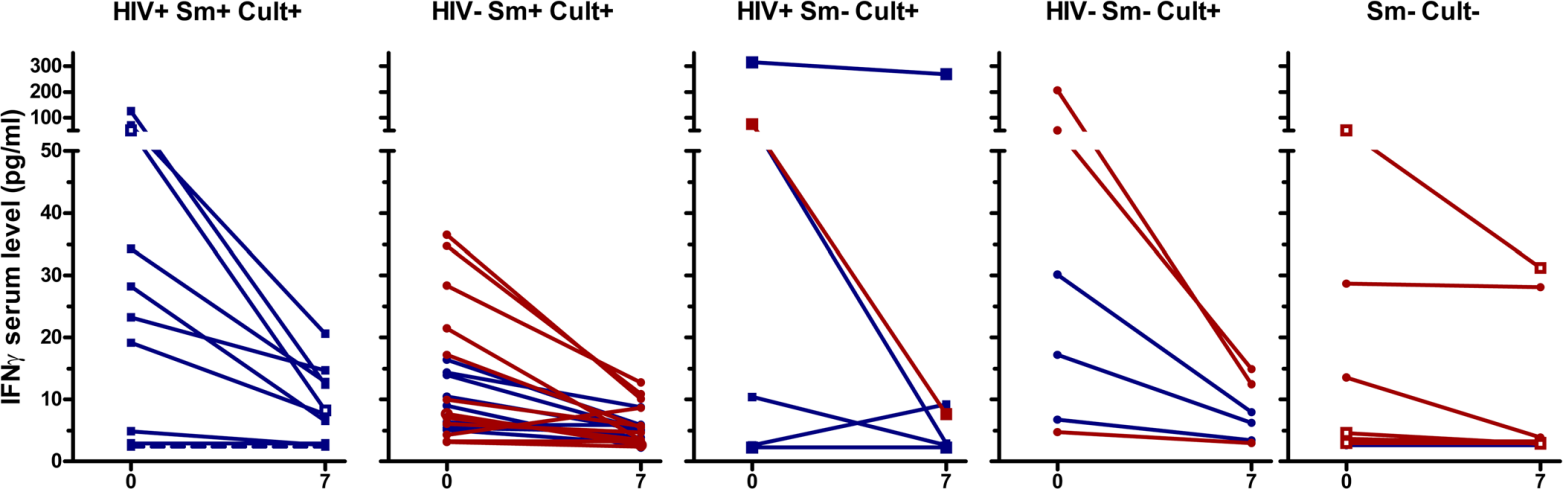
Serum IFNγ levels of TB patients at day 0 and day 7 of therapy. See legend Fig 2 for details on the panels and labels. For culture/GeneXpert positive patients p<0.001 between day 0 and day 7. For culture/GeneXpert negative patients p<0.05 between day 0 and 7.

**Fig 4:**
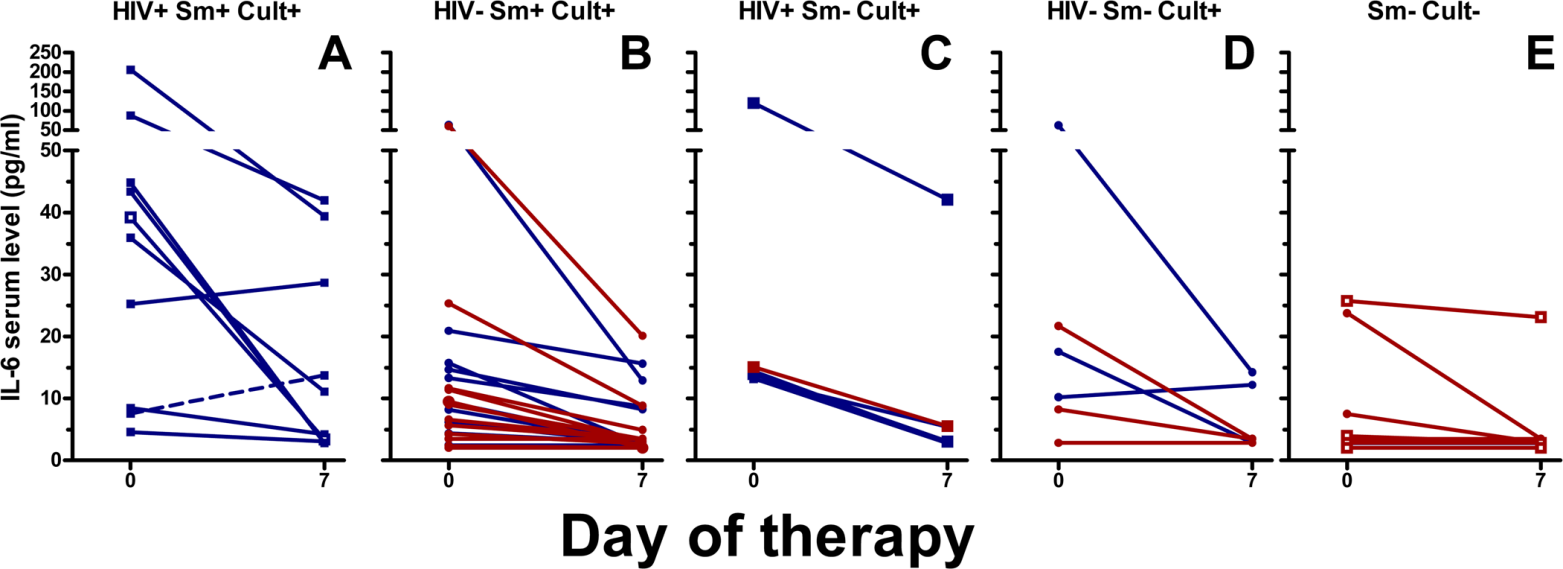
Serum IL-6 levels of TB patients at day 0 and day 7 of therapy. See legend Fig 2 for details on the panels and labels. Between day 0 and day 7, for culture/GeneXpert positive patients p<0.001; between day 0 and day 7, for culture/GeneXpert negative patients, p<0.05.

**Fig 5:**
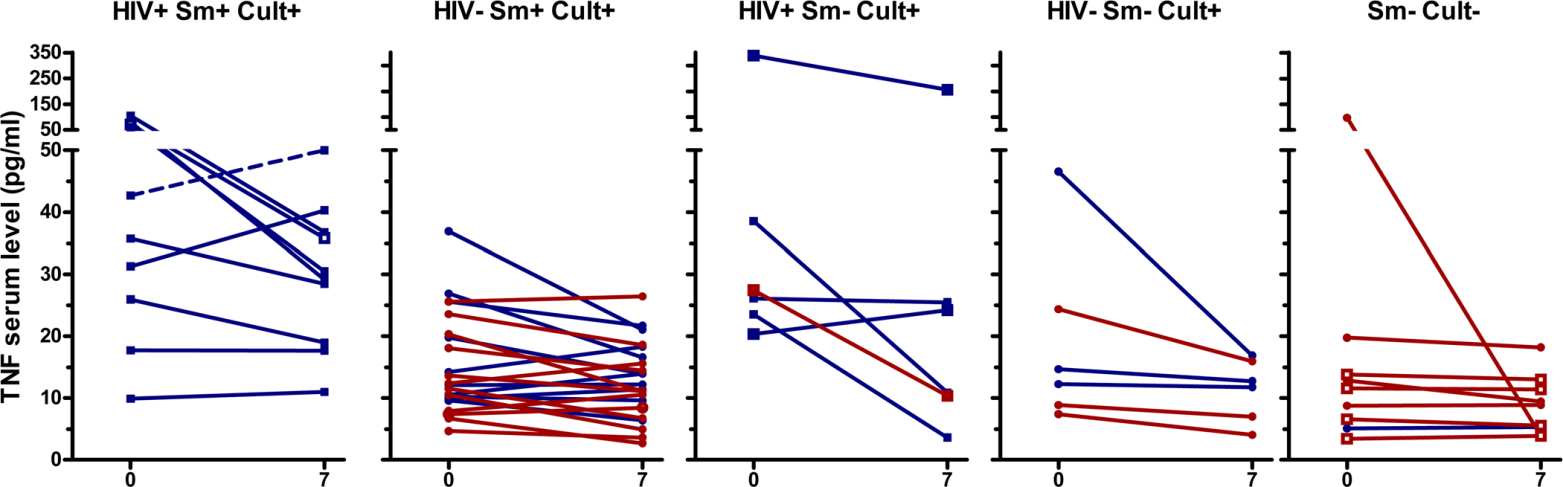
Serum TNFα levels of TB patients at day 0 and day 7 of therapy. See legend Fig 2 for details on the panels and labels. P<0.001 between day 0 and day 7; No significant differences between day 0 and day 7 for culture/GeneXpert negative patients.

**Fig 6:**
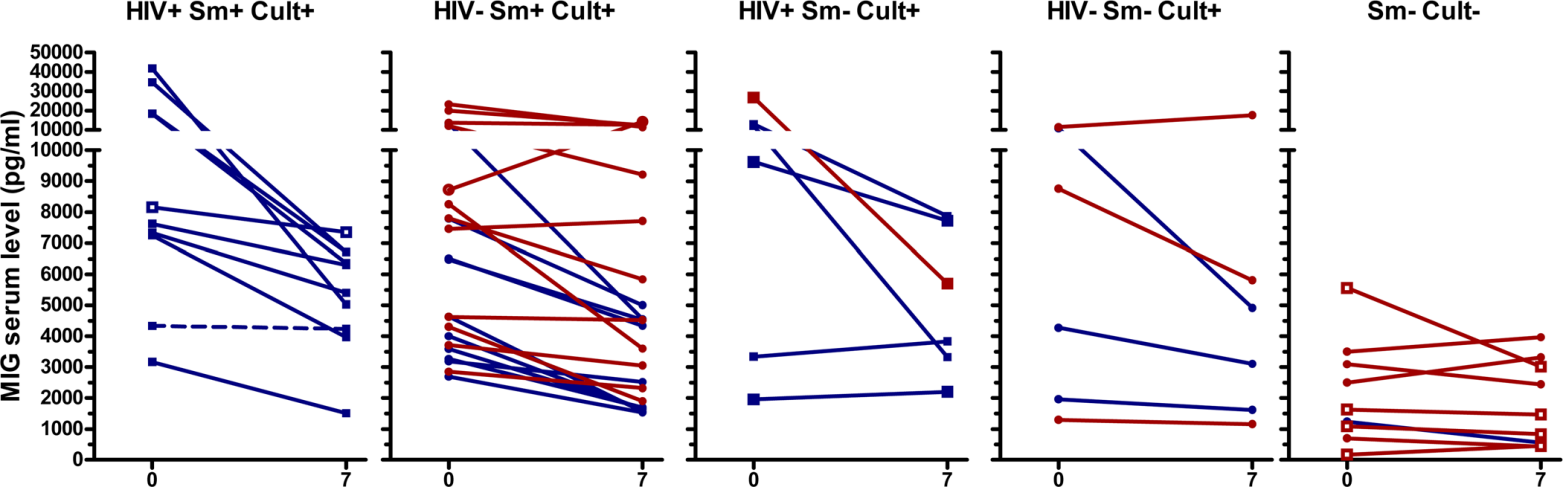
Serum MIG levels of TB patients at day 0 and day 7 of therapy. See legend Fig 2 for details on the panels and labels. P<0.001 between day 0 and day 7 for culture/GeneXpert positive patients No significant differences between day 0 and day 7 for culture/GeneXpert negative patients.

IFNγ, IL-6, and TNFα concentrations showed similar patterns as IP-10, although in a lower concentration range (Fig 3, Fig 4, Fig 5, S2 Fig, S3 Fig, S4 Fig, S5 Fig). TNFα decreased in culture/GeneXpert positive patients (p<0.001 for the overall difference between days 0 and day 7, p-values between 0.02 and 0.5 for individual smear positive and negative, HIV positive and negative subgroups), and no significant change was seen in culture/GeneXpert negative patients (p>0.05). IFNγ and IL-6 levels in culture/GeneXpert positive patients decreased between days 0 and 7 (overall both p<0.0001) and changes were weakly significant in culture/GeneXpert negative patients (p = 0.05 and 0.03, respectively). In ROC analysis of these cytokine shifts to identify patients receiving appropriate treatment, the areas under the curve (AUC) were 0.78 [95% CI 0.63–0.94], p>0.01 for IFNγ, 0.80 [95% CI 0.65–0.95], p<0.01 for IL-6, and 0.64 [95% CI 0.46 to 0.82], p>0.05 for TNFα, respectively (Panels B-D in S2 Fig). MIG serum levels in culture/GeneXpert positive patients decreased between days 0 and 5 to 7 (p<0.001; Fig 6, S6 Fig). However median concentrations had returned to enrolment values by day 14. This transient decrease in MIG was not observed in culture-negative patients (p>0.05). In ROC analysis performed as described above, AUC for the day 0-day7 change in MIG levels was 0.81 [95% CI 0.69 to 0.94], p<0.01 (Panel E in S2 Fig).

**Fig 7:**
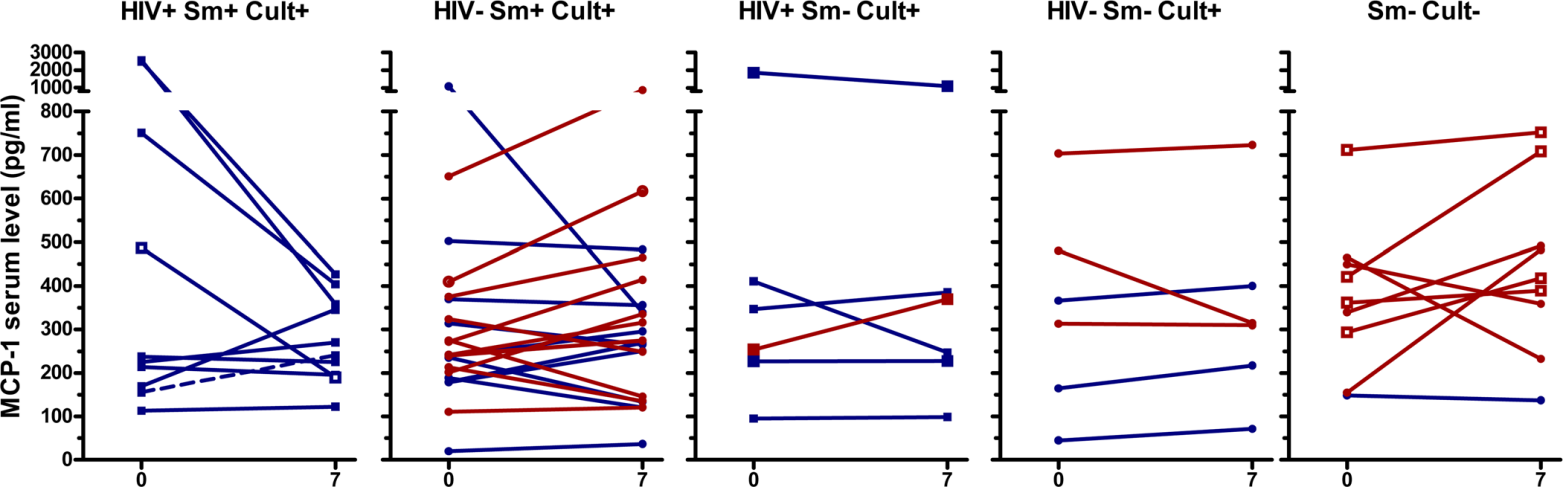
Serum MCP-1 levels of TB patients at day 0 and day 7 of therapy. See legend Fig 2 for details on the panels and labels. There are no significant differences between day 0 and day 7 for either culture/GeneXpert positive patients or culture/GeneXpert negative patients.

No changes in MCP-1 concentrations could be consistently correlated to culture/GeneXpert result (p>0.05; Fig 7, S7 Fig), nor to appropriate treatment (AUC of ROC curve day0-day7: 0.67 [95% CI 0.47 to 0.87], p>0.05, Panel F in S2 Fig).

### Serum Antibody concentrations

**Fig 8:**
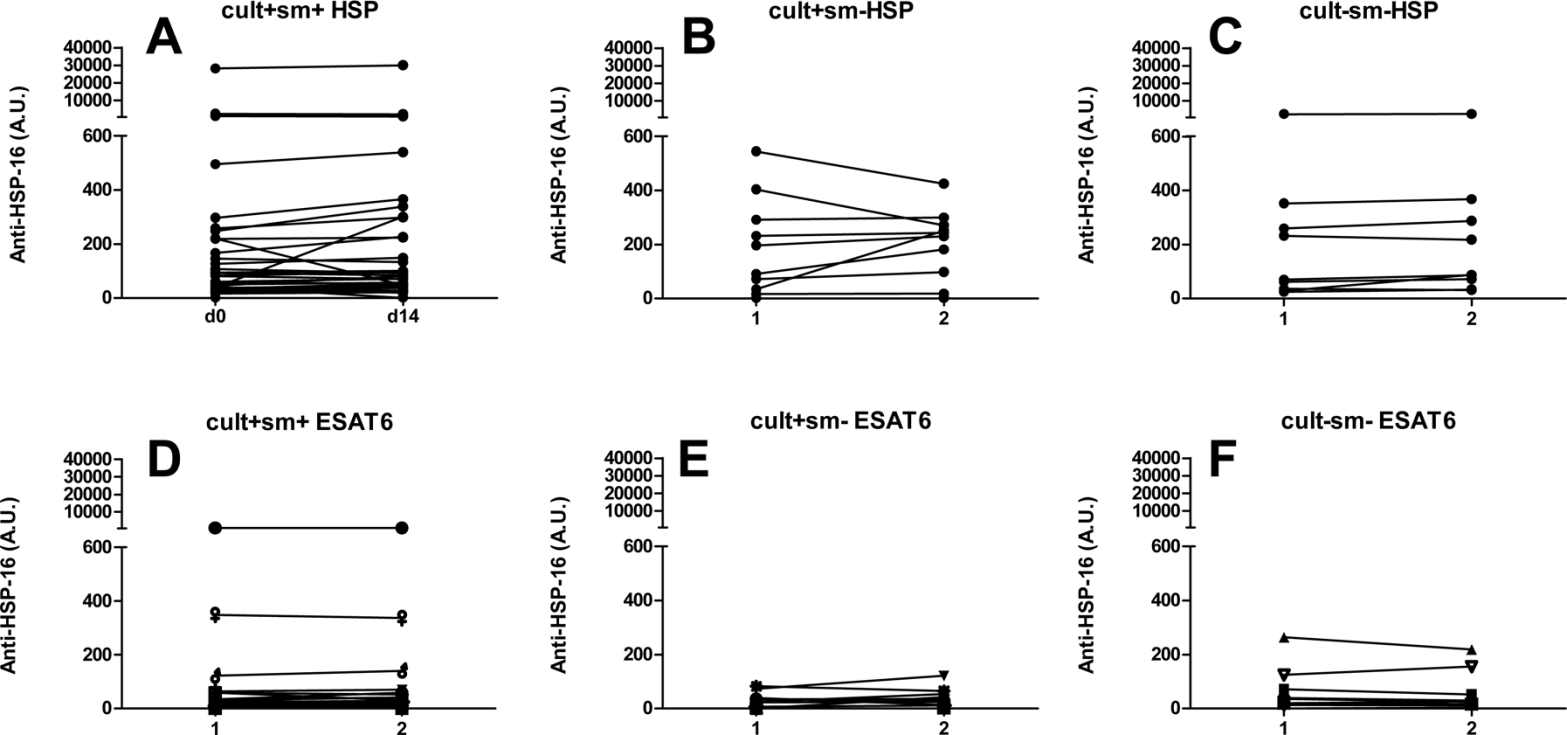
Serum antibody levels at day 0 and day 14 of therapy. Arbitrary Units (AU) of serum levels of antibodies against HSP-16 (A-C), and ESAT-6 (D-F) from a subset of TB patients. Data shown for smear positive culture/GeneXpert positive (A, D), smear negative culture/GeneXpert positive (B, E) and smear negative culture/GeneXpert negative patients (C, F). * indicates p = 0.039 between day 0 and day 14. In all other groups no significant differences were found between day 0 and 14.

There was a high variation in antibody levels against HSP-16 and ESAT-6 on enrolment (>10^3^ fold). Antibody titers remained remarkably stable within the two week follow up (Fig 8), with slightly lower HSP antibody concentrations at day 14 in culture-negative patients (p<0.039). The absolute change was small (median change 5 AU and 15 AU between day 0 and 14 in culture/GeneXpert positive and culture-negative patients respectively).

Thirty one patients were tested by lateral flow assays for serum antibodies against LAM and other TB antigens. Of those 13 (59%) of 22 culture/GeneXpert positive and two (22%) of nine culture negative patients showed a detectable band in the PROTOLAM assay at day 0 (Table 3). In three patients a change in positivity grade was identified between day 0 and day 14; two went from positive to weakly positive and one from weakly positive to negative between days 0 and 14. The TB-ST test gave detectable bands in four (18%) of 22 culture/GeneXpert-positive samples.

**Table 3:** Rapid test results at days 0 and 14 of treatment.

			PROTOLAM			TB-ST		
		Total N tested	Total positives day 0	Total positives day 14	N of patients with changed grade day 0-day 14	Total positives day 0	Total positives day 14	N of patients with changed grade day 0-day14*
Culture/GeneXpert+	total	22	13	12	3*	4	4	0
	sm+	16	10	9	3*; 1pt w to neg	3	3	0
					2pt p to w			
	sm-	6	3	3	0	1	1	0
Culture/GeneXpert-	sm-	9	2	2	0	0	0	0

Bands were graded as strongly positive, positive (p), weakly positive (w), or negative (neg). No changes in band intensity between day 0 and 14 were identified for the TB-ST, and only 3 in the PROTOLAM test.

* indicates numbers of patients (pt) with a difference in band intensity between day 0 to day 14.

## Discussion

The diagnosis of smear-negative TB is still a clinical challenge despite the development of new *in vitro* assays in the last decades. Nearly half of the patients treated for TB in low and middle income countries are treated based on clinical suspicion without bacteriological confirmation [1]. Thus, many of these patients may not have TB and are unnecessarily exposed to prolonged treatment with anti-TB drugs and remain untreated for their underlying conditions. Furthermore, newly diagnosed TB patients are in general presumed to be infected with drug-sensitive strains. Patients infected with unidentified drug resistant strains typically undergo several months of ineffective therapy before monitoring results reveal the prescribed treatment as inappropriate [12]. Therefore, there is an urgent need to develop new diagnostic tools that could confirm a clinical diagnosis of TB and identify patients potentially infected with drug resistant strains.

We described the serum kinetics of IP-10, IFNy, IL-6, MIG, TNFα, and MCP-1 before and during the first two weeks of treatment in patients with presumptive TB to explore whether cytokine responses have the potential to confirm a clinical diagnosis in patients without bacteriological confirmation and be used as early surrogate markers of treatment response to identify drug-resistant TB.

Most patients in the study with bacteriologically confirmed TB had increased baseline IP-10 concentrations. IP-10 serum levels have been previously found to be elevated in patients with TB [10,13]. However, IP-10 concentrations may also be elevated in unrelated diseases,[14] impairing specificity [15].

The early responses of the cytokines to anti-TB treatment correlated well with TB infection in a mouse model [9]. Here IP-10 serum concentrations decreased after 7 days of therapy among the majority of bacteriologically confirmed cases and a decrease of >300 pg/ml between baseline and day 7 values was observed in 75% of both smear-positive and smear-negative patients, suggesting that the pattern is independent of the number of bacilli in sputum.

HIV-TB co-infected patients are more difficult to diagnose and thus are more likely to receive empirical and potentially inappropriate TB treatment [3]. Patients co-infected with HIV had significantly higher baseline IP-10 levels than HIV-negative patients, which contrasts with previous studies reporting no statistical difference between the groups [13,16]. Despite the observed difference at baseline in our patients, TB therapy resulted in IP-10 reductions among both HIV-positive and HIV-negative patients with bacteriologically confirmed TB. It must be noted that none of the culture negatives were confirmed to be HIV coinfected thus an effect of HIV infection on the response of culture negative TB patients could not be excluded. Increased serum levels of IP-10 have been reported to return to baseline during and after TB therapy [10,13,16–18] and monitoring IP-10 after starting treatment or post treatment may be clinically informative [19] to predict cure or detect relapse [20]. In our study we demonstrated that changes in IP-10 levels in response to therapy can be detected at much earlier time points, irrespective of smear or HIV status. IP-10 concentrations were higher than all other cytokines except MIG and are therefore easier to measure than the other markers. We produced ROC curves demonstrating the ability of cytokine shifts between day 0 and day 7 of treatment to identify patients receiving appropriate treatment. Based on these analyses we were able to set criteria that classified 75% of the culture positive (including 75% of the smear-negative culture positive) patients as responding based on a threshold of 300pg/ml decrease in IP-10. All nine culture negative patients were identified as being on inappropriate treatment after seven days of treatment. It must also be noted that at least one of the non responding culture/GeneXpert positives was infected with an MDR TB strains based on subsequent DST testing. Our results thus demonstrate that IP-10 response between 0–7 days of treatment can be used for identifying patients on inappropriate treatment, who may then be prioritized for further characterization of infection and resistance profile by rapid molecular methods. Nonetheless this is a preliminary study and accurately defined criteria for response or lack of response should be established based on ROC curve analyses on larger studies including individuals with latent infection and uninfected suspects.

Although the responses of IFNγ, IL-6, and MIG after one week of treatment followed the same trend as IP-10, the accuracies of IFNγ, IL-6, and MIG kinetics for classification of TB patients as being on appropriate /inappropriate treatment were lower than for IP-10. There was also more fluctuation between time points. Changes in MCP-1 and TNFα levels with therapy were too small (Area under the ROC curves of 0.67 and 0.64 respectively) and inconsistent to have potential as markers of early treatment response.

A limitation of our study is that we did not control or monitor other conditions that may increase IP-10 levels. However, many of these conditions would not be expected to respond to TB therapy. We also did not look at any possible link between the magnitude of IP-10 responses early in treatment (or later) and treatment failure/relapse, this would be interesting to investigate this in larger studies. Full DST was only available for four patients, and there may have been cases with mono resistance that were not identified. Different drugs target different bacterial subpopulations [21] and early bactericidal activity (EBA) is higher for INH than for RIF [22]. To our knowledge there is no data on the cytokine responses of patients exposed to single drugs, but it is possible that the drug with the strongest EBA (INH) is responsible for the majority of the effect observed. The effects of individual drugs and the corresponding effect of different resistance phenotypes on the early cytokine response remain to be investigated.

The antibodies levels, as measured by ELISA and lateral flow assays, did not change during the initial 14 days of therapy. Increases in antibody titers during the early phase of therapy have been reported by some [23] but not all authors [24] and this seems to depend on the antigen [25]. Our data confirms that the utility of measuring anti-TB antibodies kinetics is limited.

The diagnosis and management of TB suspects is constrained by the intrinsic limitations in accuracy and timeliness of results with current, predominantly sputum reliant, diagnostic methods. Furthermore, in low and middle income countries, cost and infrastructure limit the availability of newer assays such as GeneXpert despite its concessionary pricing. The utility of sputum PCR is also limited for certain groups, such as patients with extra-pulmonary TB, patients unable to expectorate sputum, and in paediatric TB. A blood based approach that assesses the IP-10 response to TB treatment has the potential to supplement current strategies especially for these difficult to diagnose groups.

In conclusion, serum concentration changes of relatively non-specific host biomarkers, particularly IP-10, in response to the first week of anti-TB therapy were strongly associated with subsequent bacteriological confirmation of TB and provided early evidence of treatment response. Monitoring IP-10 response to treatment may help identify patients who are erroneously clinically diagnosed with TB or those infected with drug resistant strains on inappropriate treatment. Near patient testing of these markers using simple platforms before and one week after initiation of therapy is feasible. This approach is promising and especially useful for difficult to diagnose patients, such as smear-negative HIV-positive patients, those unable to expectorate sputum, extra-pulmonary TB, and pediatric TB patients, often treated without bacterial confirmation.

## Supporting Information

S1 Fig Relative serum IP-10 levels in Nigerian (blue) and Nepalese (red) TB patients at day 0 to day 14 of therapy. Levels shown are relative to day 0 levels (day 0 subtracted), negative values thus indicating a decrease and positive values indicating an increase compared to day 0. Panel A: Relative cytokine concentrations of HIV-positive, Smear (Sm) positive, and culture/Xpert (Cult) positive patients. The MDR-TB patient is shown with a dashed line. One smear-positive patient had missing culture (Open symbols). Panel B-D: Relative cytokine concentrations of HIV- Sm+ (B), HIV+ Sm- (C), and HIV- Sm- (D) culture/GeneXpert positive patients. E: Relative cytokine concentrations from Sm-Cult- patients, of which 4 had missing HIV result (open square symbols) and all others were HIV- (round symbols).
(EPS)

S2 Fig Receiver operating characteristic (ROC) curves showing the ability of cytokine shifts between day 0 and day 7 to identify patients receiving appropriate treatment. Patients receiving appropriate treatment were defined as those with confirmed (culture/GeneXpert positive)TB without evidence of MDR. Patients receiving inappropriate treatment were defined as those not infected with strains susceptible to the prescribed treatment, i.e. the culture/GeneXpert negative patients + MDR patients. Complete ROC statistics are presented in the Results section. * p<0.05, ** p<0.01, *** p<0.001.
(EPS)

S3 Fig Relative serum IFNγ levels in Nigerian (blue) and Nepalese (red) TB patients at day 0 to day 14 of therapy. See legend S1 Fig for details on the panels and labels.
(EPS)

S4 Fig Relative serum IL-6 levels in Nigerian (blue) and Nepalese (red) TB patients at day 0 to day 14 of therapy. See legend S1 Fig for details on the panels and labels.
(EPS)

S5 Fig Relative serum TNFα levels in Nigerian (blue) and Nepalese (red) TB patients at day 0 to day 14 of therapy. See legend S1 Fig for details on the panels and labels.
(EPS)

S6 Fig Relative serum MIG levels in Nigerian (blue) and Nepalese (red) TB patients at day 0 to day 14 of therapy. See legend S1 Fig for details on the panels and labels.
(EPS)

S7 Fig Relative serum MCP-1 levels in Nigerian (blue) and Nepalese (red) TB patients at day 0 to day 14 of therapy. See legend S1 Fig for details on the panels and labels.
(EPS)
